# From pesticides use to agroecology: Survey dataset on farming systems in the Lake Guiers area in Senegal

**DOI:** 10.1016/j.dib.2026.112577

**Published:** 2026-02-14

**Authors:** Mamadou B. Traoré, Raphaël Belmin, Rahim Ouedraogo, Paula Fernandes, Dieynaba Sall Sy, Hassna Founoune, Djibril Djigal, Mamadou Ba, Ousmane Samaké, Frédéric Feder, Aboubacry Kane, Marie-Liesse Vermeire

**Affiliations:** a UCAD, Department of plant biology, P.O. Box 5005, Fann, Dakar, Senegal; b CIRAD, UPR Recyclage et Risque, F-34398 Montpellier, France; c Recyclage et Risque, University of Montpellier, CIRAD, F-34398 Montpellier, France; d IESOL, IRD-ISRA, Bel-Air Center, P.O. Box 1386, P.C 18524, Dakar, Senegal; e CIRAD, UPR HortSys, F-34398 Montpellier, France; f HortSys, University of Montpellier, CIRAD, F-34398 Montpellier, France; g ISRA, Bureau d’Analyses Macro-Ecocomiques, P.O. Box 1386, P.C 18524, Dakar, Senegal; h SAED, Avenue Insa Coulibaly, Route de Rosso, Ngallèle, RN2, P.O. Box 74, Saint Louis, Senegal; i ISRA, Bel-Air, LNRPV, Route des Hydrocarbures, P.O. Box 3120, Dakar, Senegal; j ISRA, Centre pour le Développement de l’Horticulture, Cambérène, P.O. Box 3120, Dakar, Senegal

**Keywords:** West africa, Agricultural practices, Diagnostic, Agrochemical uses, Smallholder farmers, Irrigated area

## Abstract

The dataset was constructed from surveys of 310 farmers operating in irrigated agricultural zones surrounding Lake Guiers in northern Senegal. The survey was carried out from the 09 to the 20 of March 2023, using KoboToolbox, with a questionnaire aiming at collecting information about: 1) farmers’ agricultural practices with a particular focus on pesticides and fertilizers use; 2) their socio-economic environment; 3) their perception of changes of climatic conditions and biotic pressures within agroecosystems and production limiting factors; 4) and the level of integration of agroecological practices in their systems. Farmers were selected to represent the main agricultural systems and cover the entire lake region. The dataset provides the first comprehensive description of farming systems and agroecological practices around Lake Guiers, offering a robust basis to analyze their diversity, dynamics, and environmental performance. It can serve as both a cross-sectional baseline and a reference for future longitudinal studies, supporting econometric, ecological, and policy analyses aimed at guiding the agroecological transition and informing sustainable land and water management in sub-Sahelian contexts.

Specifications Table

**Table:** 

Subject	Social sciences
Specific subject area	Description of farmers’ agricultural activities and socio-economic environment in the Lake Guiers area, Senegal*.*
Type of data	Table, .csv format – Raw and additional simplified variables created by consolidating raw data. Readme .csv format. Questionnaire .pdf format.
Data collection	Data were collected from the 09 to the 20 of March 2023, by a team of 15 interviewers using a questionnaire that was implemented in KoboToolbox Version 2.023.04b [1]. They were cleaned using Microsoft Office - Excel (Version 16.0.14334.20440) and R software Version 4.5.1 [2]. The 310 participants to the survey were selected by a two-stage stratified random sampling to be representative of the farming systems and the studied area. The questionnaire design was built upon a model initially developed through a series of case studies conducted in Kenya, Côte d’Ivoire, Benin, Mali, and Burkina Faso, and was subsequently adapted [3,4].
Data source location	• Institution: CIRAD, ISRA, SAED, IRD • City/Town/Region: Lake Guiers area (Saint-Louis region, Dagana and Louga departments) • Country: Senegal • Latitude and longitude: between 15°54′29,34″N and 16°26′19,08″N latitudes and 15°41′44,74″W and 16°00′18,22"W longitudes.
Data accessibility	Repository name: Cirad Dataverse Data identification number: 10.18167/DVN1/C3ZMHQ Direct URL to data: https://doi.org/10.18167/DVN1/C3ZMHQ
Related research article	None*.*

## 1 Value of the Data

• These data are valuable because they provide the first comprehensive and scientifically reliable description of existing farming systems and agroecological practices in the agricultural zones surrounding Lake Guiers. Lake Guiers is a strategic area for Senegal. Supplying over 60 % of the drinking water for the capital Dakar, and also the northwest of the country (Saint-Louis, Thiès, Louga), it is central to agricultural, pastoral, and agro-industrial activities in the North of the country. Preserving this resource, as well as the diverse activities that depend on it, is a major challenge. Understanding and adapting agricultural practices is crucial in this regard. Thus, this database offers a valuable foundation for analyzing the diversity and dynamics of production systems in this key irrigated area of northern Senegal. A detailed understanding of existing farming systems can inform land-use planning, hydro-agricultural development policies, and the strategic allocation of public and donor investments.
• The dataset can be reused for econometric analyses to assess and model the socioeconomic factors shaping farmers’ technical choices, including transformation from conventional to agroecological practices in response to variable environmental drivers such as perceived climatic and biotic pressures.
• It also serves as a baseline description of farming systems at a given point in time (cross-sectional approach), thereby providing a reference for future longitudinal surveys aimed at monitoring potential transformations in farming systems.
• The dataset can be mobilized to assess the environmental performance of farming systems, for instance as a basis for life cycle assessment (LCA) studies.
• When coupled with edaphic, entomological, soil microbial community, botanical and water quality data, the dataset can help identify the ecological determinants of variability in soil, plant, water, and ecosystem health across the Lake Guiers agricultural landscape. The coupling of these complementary data may enable, among other studies, the interpretation of patterns of nitrogen and phosphorus pollution as well as pesticide residues and other forms of environmental contamination, and the identification of potential causes of biodiversity losses in ecosystems.
• Finally, the Lake Guiers dataset provides a benchmark for comparative analyses of irrigated farming systems across other sub-Sahelian agro-climatic contexts.

## 2 Background

**Fig. 1:**
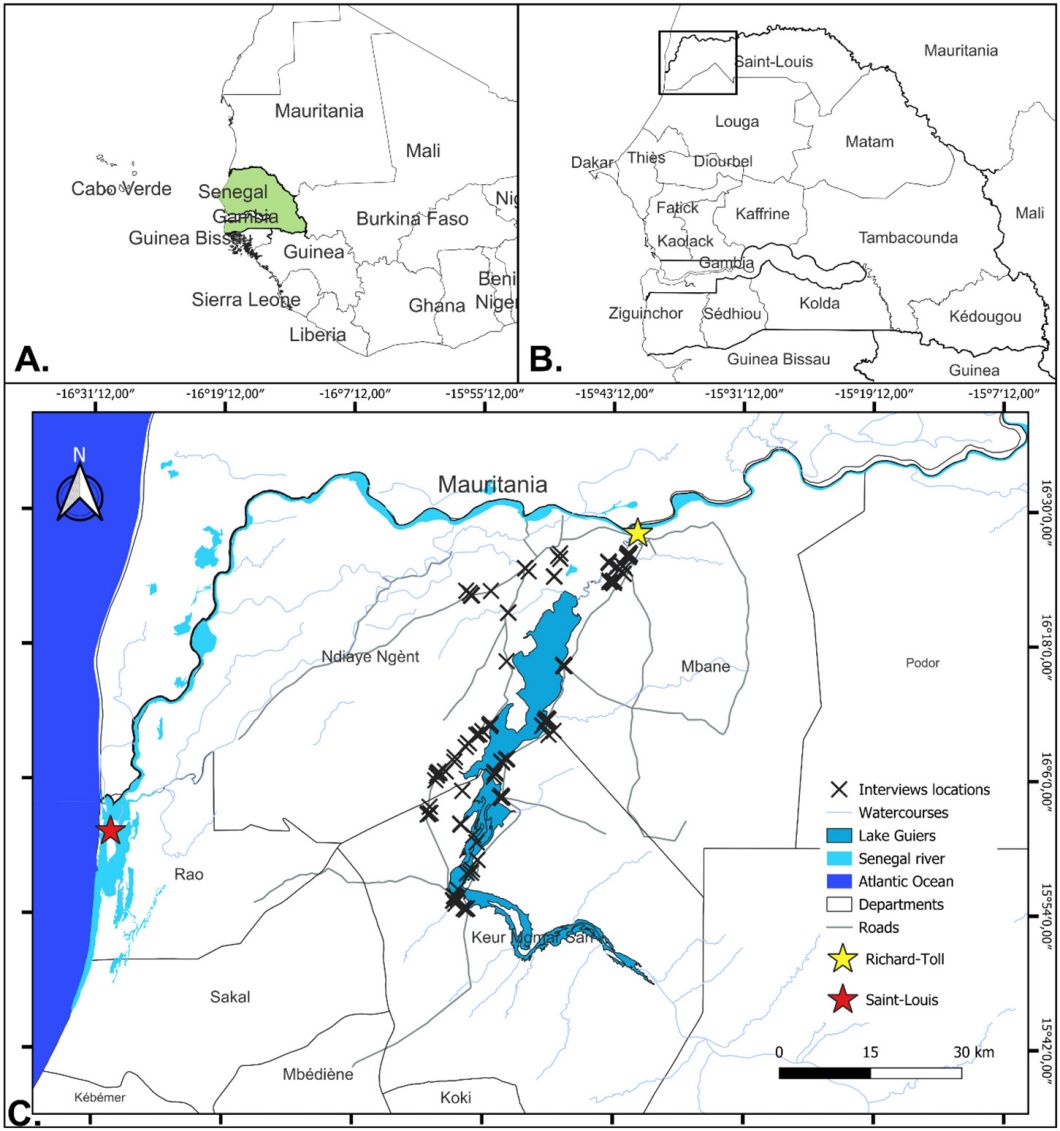
Location of the study area. A - Location of Senegal in West Africa. B - Location of Lake Guiers in Senegal. C - Lake Guiers and interviews locations.

The Lake Guiers region in Senegal (Fig. 1), covers about 30,000 hectares of cultivated land and supports activities like housing, fishing, pastoralism, fish farming, and agro-industries [5]. The lake, with a capacity of 600 million m³ [6], is vital for supplying water to the Dakar-Mbour-Thies region, which generates half of Senegal's Gross Domestic Product [7]. However, intense water use for irrigation, livestock, and drinking raises concerns about water quality [8]. Preliminary studies found that the current agricultural systems, heavily reliant on mineral fertilizers, flood irrigation and pesticides, are damaging soils and polluting the lake [5,[9], [10], [11]]. The expansion of conventional farming worsens these problems, causing a dramatic drop in fish populations and severe ecological stress [9]. To address this, a systemic agroecological transition is needed to improve soil health and restore the lake’s ecological functions, by reducing fertilizers and pesticides. This dataset was collected to characterize farming systems and factors influencing farmer decisions. The research is part of the *Santés et Territoires* project under the EU-AFD DeSIRA program, promoting a “One Health” approach to increase the health of territories via participatory *Living Labs* to co-design and test context-specific agroecological innovations.

## 3 Data Description

The files are located in a dataset named ``Diagnostic of agricultural production systems in the Lake Guiers area, Senegal'', by Traoré, Mamadou B; Belmin, Raphaël; Ouedraogo, Rahim; Fernandes, Paula; Sall Sy, Dieynaba; Ba, Mamadou; Samaké, Ousmane; Vermeire, Marie-Liesse, at the following address: https://doi.org/10.18167/DVN1/C3ZMHQ, in the Cirad Dataverse. The dataset contains six files:

• QUESTIONNAIRE_DIAGNOSTIC_FR.csv: the French version of the questionnaire used for the surveys. It includes the survey questions and response options;
• QUESTIONNAIRE_DIAGNOSTIC_EN.csv: includes the same survey questions and response options but is translated in English;
• READ_ME_BDD_FR.csv: describes, per column of the dataset, the variable name, data type, a description, and the values/units in French;
• READ_ME_BDD_EN.csv: describes, per column of the dataset, the variable name, data type, a description, and the values/units in English;
• Dataset_Diagnostic_Santes-Territoires_fr.csv: the dataset itself (answers of the farmers) in French;
• Dataset_Diagnostic_Santes-Territoires_en.csv: the dataset itself (answers of the farmers) in English.

In the dataset, each column is related to the corresponding question in the survey with the question number in the name of the column. The dataset gathers information which fall into four main categories, allowing us to have a detailed description of the agricultural system of a farmer and its socio-economic environment. These four categories were:

• **Personal and farm information (Q1-Q9 in the questionnaire)** – This category provides a description of the farmer and its sources of income. Furthermore, it contains general information on the farming system, including the fields size, the crops produced, their destinations and general agricultural practices used for soil preparation, irrigation, fertilization, crop protection, residues management and the origins of inputs.
• **Socio-economic environment of the farm (Q10-Q11)** – This category describes the relationships among farmers and between farmers and other actors such as buyers, public services and suppliers. It details the inclusion of farmers within professional organizations, the process of commercialization of their production and its implications on their practices. It gives the origins of agronomic information that farmers receive whether it is coming from media, banks, buyers, public services or suppliers.
• **Farmer perceptions of changes in its agroecosystem and innovative solutions (Q12-Q14)** – this category reports farmers uses of synthetic pesticides in particular, the evaluation of their efficiency and the awareness of their detrimental effects on human and environmental health. Then it informs about farmers' level of knowledge and usage of alternative solutions in particular agroecological practices and their willingness to adopt them. Lastly it provides farmers perceptions of climatic changes and biotic fluctuations within their agroecosystem and the way perceived changes impact their practices.
• **Detailed description of the agricultural practices in cropping season of the reference crop (Q15-Q18)** – this category provides a detailed description of all the practices from sowing to harvest to produce the most economically important crop for the farmer during the preceding cropping season. It gives precise information on the plant material used, the quantities, identity and frequency of synthetic pesticides and fertilizers used, the use of biopesticides, the irrigation frequency, the limiting factors to crop yield and the labor force employed for each farming operation in terms of number, duration and costs.

A single data type is assigned to each column including: Boolean, date, interval of number, number and text. Columns from left to right are in the same order as questions in the questionnaire. The format of the data within each column depends on the question type:

• **Columns corresponding to open-ended questions** – the column contains a value corresponding to the farmer’s answer to the question.
• **Columns corresponding to questions with a list of choices**: when only one answer can be chosen, the answer is present in one column. When several answers can be chosen, the answer is presented in two different ways: one individual column in Boolean format (yes/no) for each of the possible choices, and one column aggregating all the choices (in text format, with a semicolon separating the options).
• **Columns corresponding to a table in the questionnaire** - This is the case, for example, of questions on the application of pesticides and fertilizers. Each line of these tables in the questionnaire represents a product, its description and its use by the farmer. The answers to this type of question have been represented in several columns in the same row (one row per farmer) of the table in the questionnaire. For each product (fertilizer or pesticide) there is information about: the product name, the unit price, the dose per hectare (ha) per usage, the usage frequency per cropping season and the dose per ha per cropping season.

All data is raw (after some cleaning steps described in the next section) excepted for columns 667 to 669, 675, 812 to 818 containing summarized data: for fertilizers we calculated the total quantity used per ha for each fertilizing element (N, P and K); for pesticides, we calculated the quantities used per ha of pesticide commercial product type (herbicide, insecticide, acaricide, fungicide, nematicide) and biopesticide.

Out of the 310 farmers surveyed, 93 % were male and 7 % were female, and 77 % were over 45 years old. Farmer’s education levels were predominantly limited to koranic schooling (63 %), 9 % had no formal education, 14 % had dropped out at primary school, 12 % had completed secondary school, and 2 % has attended university.

The majority of farms (62 %) had a size ranging from 1 ha to 5 ha, while 30 % were <1 ha and 8 % were larger than 6 ha. Cassava and rice are the most important crops in the survey, cultivated by 33 and 28 % of farmers, respectively. These are followed by peanut (11 %), watermelon (9 %), sweet potato (7 %), onion (7 %) and other vegetables (including tomato, lettuce, okra, banana, cabbage, melon and chili) which are cultivated by <5 % of farmers.

**Fig. 2:**
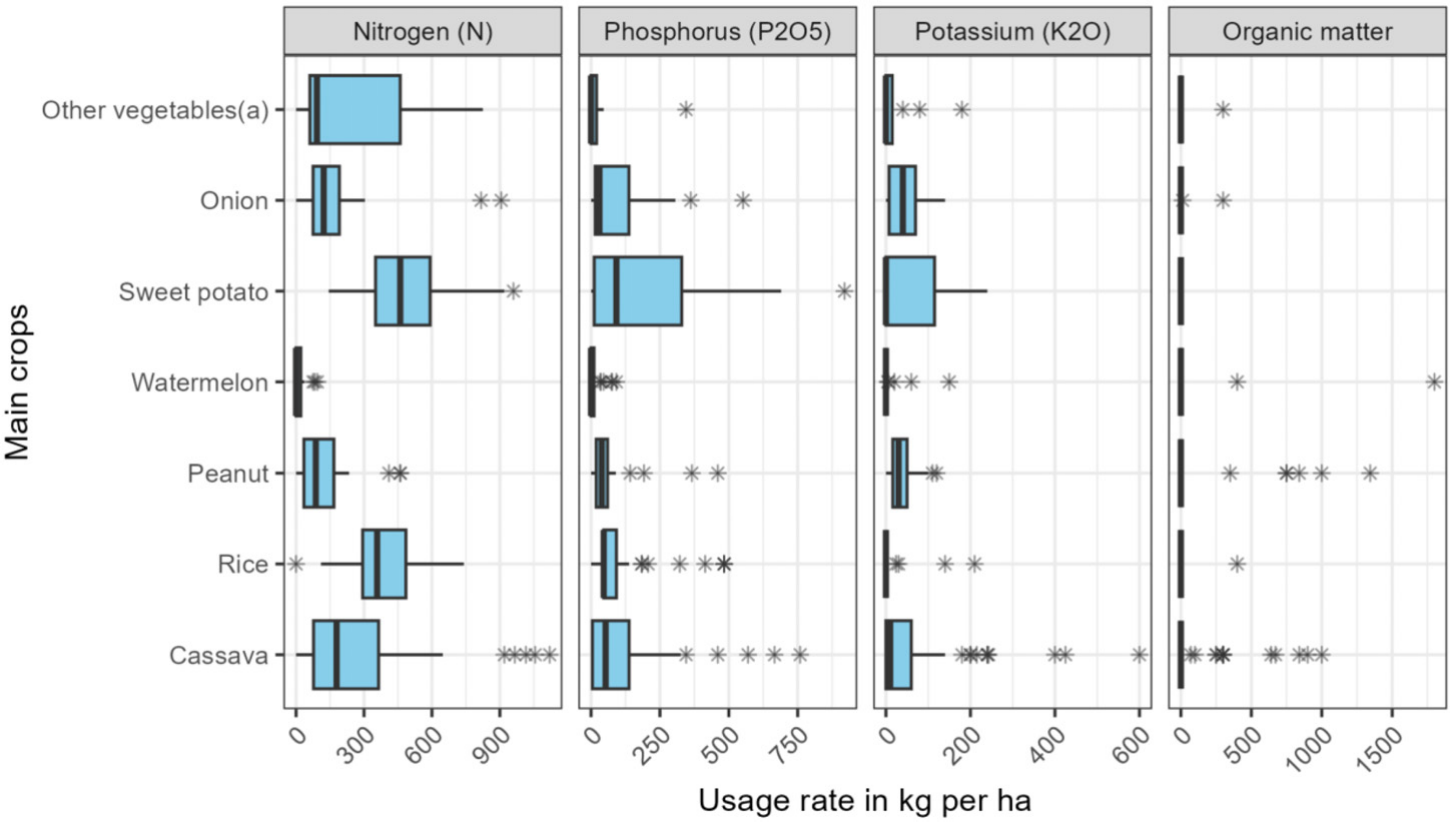
Usage rates of fertilizers in one cropping season of the main crop (*n* = 310). (a) other vegetables include Tomato, lettuce, okra, banana, cabbage, melon and chili.

The usage rates of Nitrogen, Phosphorus, Potassium and organic matter per main crop cycle are shown in Fig. 2.

**Fig. 3:**
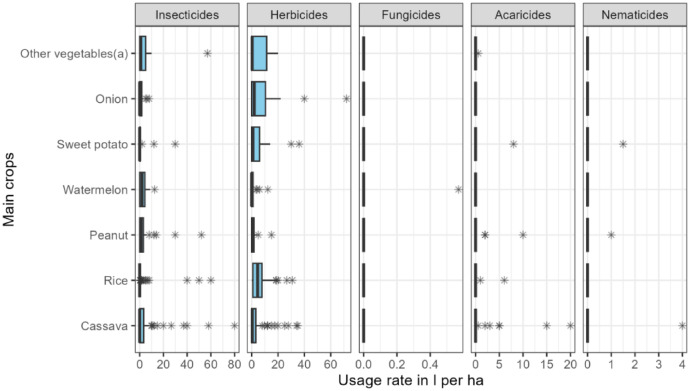
Usage rates of pesticides in one cropping season of the main crop (*n* = 310). (a) other vegetables include Tomato, lettuce, okra, banana, cabbage, melon and chili.

The usage rates of pesticides (insecticides, herbicides, fungicides, acaricides, nematicides) per main crop cycle are shown in Fig. 3.

Farmers knowledge, use and willingness to use of agroecological practices are presented in Table 1.

**Table 1:** Agroecological practices knowledge, use and willingness to use among farmers ( % of farmers).

Agroecological practices	Willingness to use	Knowledge	Already using
Intercropping	31 %	58 %	51 %
Crop rotation	42 %	91 %	74 %
Trees integration	1 %	26 %	5 %
Augmentarium	24 %	2 %	0 %
Crop auxiliaries	16 %	6 %	1 %
Commercial biopesticides	55 %	31 %	4 %
Homemade biopesticides	55 %	35 %	5 %
Compost	48 %	44 %	7 %
Cover crop	10 %	6 %	1 %
Strip cropping	9 %	11 %	7 %
Poultry manure	24 %	42 %	19 %
Net	17 %	11 %	2 %
Cattle manure	26 %	67 %	52 %
Hedges	23 %	33 %	27 %
Fallow	48 %	88 %	61 %
Beneficial indigenous microorganisms	28 %	2 %	0 %
Artificial pond	5 %	1 %	0 %
Mulching (Imported)	15 %	17 %	6 %
Mulching (residues left on the field)	9 %	10 %	2 %
Crop nursery with alveolar trays	46 %	49 %	12 %
Attractive plants	31 %	7 %	0 %
Host plants to crop auxiliaries	27 %	6 %	0 %
Nematicide plants	37 %	9 %	1 %
Repellent plants	33 %	7 %	2 %

## 4 Experimental Design, Materials and Methods

### 4.1 Survey questionnaire

The survey questionnaire items were developed based on previous studies aimed at describing agricultural production systems and levels of agroecological transition across West African countries [3,4]. The questionnaire was designed to collect information across the four categories mentioned above: personal and farm information, socio-economic environment of the farm, farmer perceptions of changes in its agroecosystem and innovative solutions, detailed description of the agricultural practices in cropping season of the reference crop. The questionnaire used for the survey is presented in English and in French in the Cirad Dataverse.

### 4.2 Sampling

A two-stage stratified random sampling of farms was used to ensure representativeness of the main municipalities surrounding Lake Guiers and the diversity of existing cropping systems, including cassava–sweet potato, vegetable production, rice, rainfed crops, peanut, and agribusiness systems (Table 2). The first stage of sampling was at the cropping system level and the second stage was within municipalities.

**Table 2:** Repartition of interviewees within cropping systems and municipalities.

**Sector**	**Municipality**	**Cropping system types**					**Total**
		Cassava-Sweet potato	Vegetable crops	Rice	Rainfed crops	Peanut	

East Lake	Ndombo	5	30	30	5	0	70
	Mbane-Syer	20	10	0	10	30	70
West Lake	Colonat-pakh	5	5	30	0	0	40
	Gnith	10	10	0	10	15	45
	Gnith-Keur Momar Sarr	15	5	0	10	15	45
Low Ferlo	Low Ferlo	5	0	0	25	0	30
Total		60	60	60	60	60	300

The sources used to draw up the stratified random sample included (i) databases from the National Service for the Development and Exploitation of the Senegal River Delta Lands (SAED), (ii) registers from the Senegalese Institute for Agricultural Research - Saint-Louis Agricultural Research Center (ISRA-CRA), (iii) a cropping system typology built by our team through preliminary survey [8], as well as (iv) lists provided by Economic Interest Grouping (GIE) chiefs and communal authorities. The following selection criteria were systematically applied:

• Balanced geographical distribution all around the lake;
• Representativeness of the different profiles of previously identified cropping systems;
• Exclusive inclusion of active farm managers;
• Avoidance of bias by not limiting selection to so-called "leader" or "pilot" producers;
• Maximization of women's participation.

The sample covers a total of 300 farms with 60 farms per cropping system type (Table 2) and 10 farms owned by women were added as they were underrepresented in the initial sampling.

### 4.3 Preparation of survey and training of the interviewers

A four-day preparation mission was devoted to organizing the survey campaign and training interviewers. Fifteen students from the Gaston Berger University (UGB), were trained to use KoboToolbox for digital input of questionnaires, to understand the content of the questionnaire and to harmonize their translation into the local language (mainly Wolof), therefore avoiding translation bias. The training program was structured in three stages:

• Theoretical classroom session devoted to presenting the objectives of the survey, the questionnaire and clarifying expectations for each section;
• Field visits for practical exercises;
• Debriefing session to adjust collection methods in the light of feedback.

### 4.4 Questionnaire testing and optimization

The questionnaire was pre-tested with six farmers in three locations (Mbane, Keur Momar Sarr, West Lake). These tests were carried out by two interviewers working separately, followed by debriefing sessions. The results showed that the digital format with KoboToolbox was clearly superior to the paper format in terms of speed and ease of input. Adjustments were then made to the questionnaire following these tests, to optimize its ergonomics and improve the efficiency of the collection process.

### 4.5 Survey implementation

The survey was carried out from the 09 to the 20 of March 2023, using KoboToolbox Version 2.023.04b [1]. Data collection took place over a period of six to seven days, with a target of 20 surveys per interviewer (i.e., around 3 surveys per day). In order to guarantee quality supervision, the first two days in the field were conducted in pairs, allowing less experienced interviewers to be accompanied. The interviewers organized their own appointments with farmers, optimizing travel and interview planning. Data collection was the subject of in-depth methodological preparation aimed at ensuring the quality and reliability of the information gathered, from the design of the questionnaire to the execution of the surveys in the field. Supervision of the interviewers during the survey was ensured by:

• Remote monitoring: daily check of data entered on the Kobo server, enabling real-time readjustment if necessary.
• Field accompaniment by SAED agents.

### 4.6 Data export, cleaning and quality control

After the survey, data were exported from the KoboToolbox to csv format, and cleaned depending on the type of question they were related to.

• **Open-ended question** - The answer is specific to each farmer, and the format of the information entered on the tablet depends on the interviewer. Answers to this type of question have been corrected for spelling mistakes, formatting (by replacing spaces and apostrophes with underscores) and removing special characters (especially letters with accents). Similar answers formulated or entered in different ways have been harmonized as much as possible.
• **Questions with a list of answers and only one answer can be chosen** - For these questions, the farmer can only give one answer from the list of answers proposed in the questionnaire. The answers to this type of question were already directly encoded in the KoboToolbox, so no additional processing was required.
• **Questions with a list of answers and several answers can be chosen** - The farmer can choose several answers from the list. These answers did not require any additional processing.
• **Questions with numerical values** – For example, the questions about uses of pesticides and fertilizers. The values of these answers were verified and corrected on the basis of references within the area of study according to the SAED agents, farmer testimonials and web searches.

In total, 16 % of all entries in the dataset are NAs. They designate answers unknown by the farmer, answers they were not willing to provide or answers to questions non applicable to their case.

## Limitations

As with most survey-based research, several limitations should be acknowledged. First, the data are derived from **self-reported responses**, which are inherently subject to individual perceptions and interpretations. This introduces risks such as **inaccuracies in recall**, where respondents may not remember past events or experiences precisely, and **response fatigue**, which can reduce attention and lead to incomplete or less thoughtful answers, especially in longer questionnaires.

Second, **socio-cultural factors** may have influenced the way participants understood and responded to survey questions. Differences in cultural norms, social desirability, and local contexts can bias responses, either by encouraging conformity to perceived expectations or by discouraging disclosure of sensitive information.

Third, there is the possibility of **respondent opportunism**, where individuals may provide answers that they perceive to be advantageous to them, rather than strictly accurate, particularly in surveys linked to access to services, benefits, or evaluations.

Finally, some questionnaire items relied on technical concepts related to agroecological practices and crop health, which may have exceeded the average level of formal education typically found in rural Senegalese contexts. Although the questionnaire was pilot-tested, administered by trained Wolof-speaking interviewers, and developed with the support of SAED, the translation and explanation of certain scientific concepts may still have led to variability in interpretation and potential **response bias**. Furthermore, the questionnaire was designed to be reusable across different West African contexts, which required a balance between local semantic adaptation and cross-site comparability. While this approach supports regional relevance, it may have limited the extent to which questions could be fully tailored to the specific linguistic and cultural context of the study area.

Measures were taken to mitigate the impacts of these limitations, for instance the possibility to take a rest during the interview as to avoid fatigue and to allow respondents to recall information. Additionally, the great number of interviews reduces the bias due to inaccurate information as well as the *a posteriori* verification of these information likelihood based on a literature review. Nevertheless, these limitations underscore the need for caution in the interpretation and generalization of the findings. While survey data provide valuable insights, the results should be viewed as indicative rather than definitive, and ideally complemented with additional methods of data collection to strengthen validity.

## Ethics Statement

All participants gave informed consent prior to participation in accordance with the Declaration of Helsinki. Respondents were informed about the purpose of the survey, the voluntary nature of participation, and their right to withdraw. Consent included permission for anonymized data to be archived and reused for research purposes. All personal identifiers were removed from the dataset prior to deposition; the shared dataset contains only non-sensitive, anonymized records and aggregated variables that prevent re-identification. Data processing and sharing comply with applicable data protection regulations including the EU General Data Protection Regulation (GDPR). These statements are testified by SAED, the local semi-public organization responsible for development in the area, provided in the supplementary file.

## CRediT Author Statement

**Mamadou Traoré:** Data Curation, Writing - Original Draft, Writing- Review and Editing, Visualization. **Raphaël Belmin:** Conceptualization, Methodology, Data Curation, Writing - Original Draft, Writing- Review and Editing. **Rahim Ouedraogo:** Conceptualization, Methodology, Data Curation, Writing - Original Draft, Writing- Review and Editing. **Paula Fernandes:** Conceptualization, Methodology, Writing- Review and Editing. **Dieynaba Sall Sy:** Writing - Review and Editing. **Hassna Founoune:** Writing - Review and Editing. **Djibril Djigal:** Writing - Review and Editing. **Mamadou Ba:** Writing - Review and Editing, Project administration. **Ousmane Samaké:** Writing - Review and Editing, Project administration. **Frédéric Feder:** Writing - Review and Editing. **Aboubacry Kane:** Writing - Review and Editing. **Marie-Liesse Vermeire:** Conceptualization, Methodology, Data curation, Validation, Writing - Review and Editing, Supervision.

## Data Availability

DataverseDiagnostic of agricultural production systems in the Lake Guiers area, Senegal (Original data)

## Appendix Supplementary materials

Image, application 1
