# Supplementary material for: From pesticides use to agroecology: Survey dataset on farming systems in the Lake Guiers area in Senegal
Source: Data Brief. 2026 Feb 14;65:112577. doi: 10.1016/j.dib.2026.112577 (PMC12950485; doi:10.1016/j.dib.2026.112577)
Supplement: Supplementary file 1 [file mmc1.pdf]

SOCIETE NATIONALE D'AMENAGEMENT ET D'EXPLOITATION DES TERRES DU DELTA DU FLEUVE SENEGAL ET DES VALLEES DU FLEUVE SENEGAL ET DE LA FALEME (SAED)

24/11/2025

Subject: Confirmation of ethical approval, consent, and lawful collection of the dataset titled "Survey dataset on farming systems in the Lake Guiers area in Senegal"

Dear Editors,

I am writing in my capacity as head of agricultural advisory and professionalization support division of SAED to confirm that the dataset entitled "Survey dataset on farming systems in the Lake Guiers area in Senegal" was collected by CIRAD, ISRA, IRD and SAED between the 09 and the 20 of March 2023 as part of the project "*Santés et Territoires*".

Ethical approval: According to the internal policies of SAED and national regulations, the study protocol did **not require review or approval by a formal ethics committee**, as it involved only voluntary, anonymous survey participation and did not collect sensitive personal or health information. This determination was verified by SAED in February 2023.

Informed consent: All participants (farmers) were provided with information about the study's purpose, data uses, and their rights. Participants provided verbal informed consent, procedures for obtaining oral consent are documented. Consent included permission for anonymized data to be archived and shared for research reuse.

Data protection and GDPR: Personal data were handled according to applicable data protection laws including the EU GDPR. Personal identifiers have been removed prior to any public deposition; the dataset deposited contains only anonymized data as per agreed procedures.

Custody and provenance: SAED confirms it is the lawful data custodian and has the authority to deposit the dataset and grant access for research purposes.

We certify that the data were collected in full accordance with institutional and legal standards for research involving human participants, and that participant privacy and consent were respected.

Yours sincerely,

MAMADOU BA
HEAD OF AGRICULTURAL ADVISORY AND PROFESSIONALIZATION SUPPORT DIVISION
SAED
24/11/2025
TEL: 77 667 92 76 / MAIL: bamamadou.15@gmail.com
